# Antimicrobial, anti-cancer, anti-filarial and anti-inflammatory activities of Cowabenzophenone A extracted from the endophytic fungus *Aspergillus terreus* isolated from a mangrove plant *Bruguiera gymnorrhyza*

**DOI:** 10.1080/21501203.2019.1707722

**Published:** 2019-12-27

**Authors:** Kushan M. Ukwatta, Jennifer L. Lawrence, C. D. Wijayarathne

**Affiliations:** a Department of Chemistry, University of Colombo, Colombo, Sri Lanka; b Departments of Chemistry and Earth, Ocean and Atmospheric Science, University of British Columbia (UBC), Vancouver, Canada; c Department of Research and Development, AMEDA Diagnostics, Austria

**Keywords:** Cowabenzophenone A, *Aspergillus terreus*, *Bruguiera gymnorrhyza*, endophytic fungus, anti-inflammatory

## Abstract

Cowabenzophenone A was isolated from an endophytic fungus Aspergillus terreus isolated from a mangrove plant Bruguiera gymnorrhyza. The structure was determined by analysis of 1D and 2D NMR spectra and mass spectrometric data as a tetracyclo[7.3.3.33,11.03,7]tetradecane-2,12,14-trione skeleton. When the compound was tested in vitro for its ability to inhibit inflammations, α-glucosidase inhibitory activities and its antimicrobial properties, it showed an anti-inflammatory activity amounting to an IC50 of 12.1 µg/mL, α-glucosidase inhibitory activity, with IC50 values of 7.8 ± 0.5 μM, and antibacterial activity against Bacillus subtilis TISTR 088, and Bacillus cereus TISTR 688 with MIC values of 1 μg/mL and 2 μg/mL respectively. The compound showed cytotoxicity against HCT 116 colon cancer cell line with an IC50 value of 10.1 ?M, and also showed a considerably high potential towards anti-filarial activities by resulting MIC, IC50 and LC50 values of 0.358 ± 0.02 mg/mL, 0.708 ± 0.021 mg/mL and 3.89 ± 0.18 mg/mL, respectively, in comparison to the standard drug Ivermectin (IVM).

## Introduction

There is a need to search for novel drug-leads to fight diverse complications in health, because infectious diseases are still a global problem due to development and spread of drug-resistant pathogens (Anaissie et al. 1996). Novel anti-cancer drugs leads are in massive demand due to high worldwide mortality (Parkin et al. 1999). Over the last few decades, endophytic fungi have been recognised as a rich source for pharmacologically active metabolites. Sri Lanka has a high rate of endemic speciation in both plants and microbes (Gunatilleke and Gunatilleke 1990), which offers great potential for the discovery of plant-associated microbes that can produce new natural products with medicinal or agricultural importance (De Silva et al., 2011; Chandra 2012).

On this scheme to identify and isolate novel drug leads, our research team had successfully obtained many compounds from endophytic fungi resident in terrestrial and aquatic plants. Biological activities associated with mycoleptodiscin B, isolated from the endophytic fungus *Mycoleptodiscus sp*., (Dissanayake et al. 2016b) chaetoglobosin A and C, produced by the endophytic fungus *Chaetomium globosum*, (Dissanayake et al. 2016a) equisetin, from endophytic *Fusarium sp*. and solanioic acid, an antibacterial-degraded steroid produced in culture by the fungus *Rhizoctonia solani* are some of them. (Ratnaweera et al. 2015b)

As a part of this ongoing programme aimed at discovering bioactive secondary metabolites of endophytic fungi obtained from Sri Lankan mangrove plants (Centko et al. 2014), the extracts of laboratory cultures of the endophytic fungi isolated from *Bruguiera gymnorrhyza* were investigated.

Mangroves are the most extensively distributed habitat in the Jaffna lagoon area and are found along lagoon borders, estuaries and along the coastline of islands (Myers et al., 2000; Mittermeier et al. 2005). Two types of mangrove communities are recorded in this area, namely riverine mangroves and fringing mangroves. A total of 13 true mangrove species and 18 mangrove-associated species have been reported from this lagoon and neighbouring islands. (Karunathilake 2003)

*Bruguiera gymnorrhyza* belongs to the *Rhizophoraceae* family. Trees would grow up to about 10 m in height and possess knee roots but lack prop roots. New growth of green leaves becoming grey, glossy and hairless. (Perera and Amarasinghe 2018) Fungi of the genus *Aspergillus* were reported to produce a high variety of secondary metabolites, exhibiting diverse and remarkable biological properties such as antifungal, anti-tubercular, anticancer, antibacterial and farnesyl protein transferase inhibitory activities (Coles et al. 2002).

Therefore, as a promising source of naturally bioactive metabolites, *Aspergillus terreus* has attracted much attention. *Aspergillus terreus* has usually been found as a saprophytic fungus, producing mycotoxins such as patulin and cytochalasin E (Dong et al. 2008). Different species of *Aspergillus* grown in different media had given rise to distinct types of compounds. Extracts of *Aspergillus terreus* grown in liquid culture had given rise to several pyrones such as ampelopyrone, sulphated derivatives of macrosporin and 3-O-methylalaternin (Yates et al. 2012). In addition to these compounds, lactones such as methyltriacetic lactone had also been isolated (Yeasmin and Shamsi 2013). Previous results, as well as the findings in the present investigation, indicate that fungus *Aspergillus terreus* as an interesting source for new bioactive metabolites.

## Materials and methods

### Collection of plant material

Fresh healthy leaves, stem, flowers, bark and fruits of *Bruguiera gymnorrhyza* were collected from Jaffna lagoon, Northern Province, Sri Lanka (90 50ʹ 0 N and 790 50ʹ 0 E to 90 20ʹ 0 N and 800 30ʹ 0 E; 1,087,208 N and 372,066 E to 1,031,744 N and 445,094 E). The collection was made in October 2014, transported to the laboratory in a tightly sealed Zip bag and stored at room temperature (30°C) under humid conditions. The identity of the plant was confirmed and authenticated by comparison with the voucher specimen in the National Herbarium at Royal Botanical Gardens, Peradeniya, Sri Lanka. The voucher specimen (No. UOC/NPL/KUBG12) of the plant used for the current study was deposited in the Pathology Laboratory, Department of Plant Sciences, University of Colombo, Sri Lanka. Collected plant parts were used for isolation of endophytic fungi within 16 h after collection.

### Isolation of endophytic fungi from the collected plant specimens

Samples were cleaned under running tap water and then air-dried. Plant parts were cut into approximately 5 cm long pieces, and the parts of the leaves, flowers, roots and stem bark were surface sterilised by immersing in 70% ethanol for 1 min, 5% sodium hypochlorite solution for 1 min and sterile distilled water for 1 min. The surface sterilised leaves, flowers, roots and stem bark were cut into 0.5 cm^2^ pieces using a sterile blade and placed separately on potato dextrose agar (PDA) medium under sterile conditions and incubated at 30°C for 21–27 days until the emergence of hyphae. Isolation of pure cultures was achieved from the original growths following serial inoculation method.

### Identification of fungal isolates

Isolation of genomic DNA from the mycelia of each fungal isolate was carried out according to the published protocol of (Kariyawasam et al. 2012). The ITS region of the isolated genomic DNA was amplified by polymerase chain reaction (PCR) using the forward primer ITS 1 (5ʹTCCGTAGGTGAACCTGCGG3ʹ) and the reverse primer ITS 4 (5ʹTCCTCCGCTTATTGATATGC3ʹ) under the conditions, initial denaturation of 5 min at 94°C, followed by 35 cycles of 30 s at 94°C, 1 min at 55°C and 2 min at 72°C, with a final extension of 7 min at 72°C (Diaz et al. 2012). The amplified DNA was sequenced and BLAST analysed [National Centre for Biotechnology Information (NCBI)]. The acquired gene sequences were submitted to the NCBI GenBank database and accession numbers were obtained.

### Extraction of crude extracts

Each single endophytic fungal isolate was grown in small scale on PDA (15 plates – 100 × 15 mm). The solid medium with the mature fungi was cut into small pieces and extracted into ethyl acetate. The ethyl acetate extract was concentrated by evaporating the solvent under reduced pressure at room temperature to obtain crude organic extracts.

### Antimicrobial activities of the crude extracts

The antibacterial and antifungal activities of the crude extracts were determined using disc-diffusion assays. Selected fungus (KUBGACJ-14) which expressed the highest antimicrobial activity was grown in large scale, on sterile PDA in 600 petri dishes (size, 100 mm × 120 mm) for 16 days at 30°C, and this culture was extracted thrice with EtOAc (3 x 2 L). The combined extracts were filtered and were concentrated *in vacuo*.

Crude extracts were assayed for antimicrobial activity against Gram-positive bacteria, *B. subtilis* (UBC 344), *S. aureus* (ATCC 43,300) and methicillin-resistant *S. aureus* (MRSA, ATCC 33,591) at 10 μg/disc and two Gram-negative bacteria, *Escherichia coli* (*E. coli)* (UBC 8161), *P. aeruginosa* (ATCC 27,853) at 100 and 25 μg/disc. The crude extracts were qualitatively assayed against pathogenic fungi *C. albicans* (ATCC 90,028), *F. oxyporum* (UBC 3512), *R. microporus* (UBC 6314), *C. gloeosporides* (UBC 9312) and *A. niger* (UBC 6514) at 50.0 μg/disc.

The major compound in the extract was assayed for antimicrobial activity against the same Gram-positive, Gram-negative bacteria and pathogenic fungi except for *F. oxyporum* (UBC 3512), *R. microporus* (UBC 6314) and *A. niger* (UBC 6514) to obtain the minimum inhibitory concentrations (MICs). The MIC values were determined using broth micro-dilution method according to National Committee for Clinical Laboratory Standards 2003. The MIC was taken as the lowest concentration with more than 90% growth inhibition by measuring optical density. The commercial antimicrobial agents, polymyxin B for *B. subtilis, E. coli* and *P. aeruginosa*, rifamycin for *S. aureus* and MRSA, amphotericin for *C. albicans* and *C. gloeosporioides* were used as positive controls.

### Isolation and structure elucidation of bioactive compounds

The crude extracts were next subjected to bioassay-guided fractionations in order to isolate the active compounds. The fractionation schemes were preceded from size exclusion chromatography (LH20) and polarity-based separation techniques (Silica columns and C18 Sep pak) by confirming the consistency of bioactivity using the bioautography technique. After every separation scheme, each individual extract was further guided by the examination of their thin layer chromatography (TLC) behaviour. Structure elucidation of the isolated active compound was done by using spectroscopic data obtained from UV, IR, and NMR (1D & 2D) spectroscopy.

To isolate the principal bioactive component from the complex mixture of the fungal extract, an advanced series of bioassay-guided purification steps were performed. The crude extract (720 mg) was first subjected to solvent/solvent partitioning between hexane and MeOH/H_2_O, 7:1 (1:1 300 mL of each) and left for 12 h; after the separation of the hexane layer, the polarity of the aqueous layer was increased to MeOH/H_2_O, 2:1 by addition of H_2_O and extracted with CHCl_3_ (300 mL×2). The CHCl_3_ layer was separated and the aqueous layer was concentrated at reduced pressure and was partitioned between H_2_O and EtOAc (200 mL×3). All four fractions (MeOH, H_2_O, EtOAc and CHCl_3_) were tested for bioactivity and continued with the combined CHCl_3_ and EtOAc soluble fractions which had retained bioactivity. Next, the combined fraction (91 mg) was eluted through a Sephadex LH-20 size exclusion chromatography (2.5 × 175-cm column) with MeOH/CHCl_3_; 5:4 as the eluting solvent. Resulting fractions were combined according to the thin layer chromatography (TLC) profiles and the combined fractions were tested for antibacterial/antifungal activity. The most active fraction (27 mg) was run on a normal phase silica (2.5 × 90-cm) column using gradient elution (10% to 60% MeOH: CH_2_Cl_2_). The resulting active fraction (21.2 mg) was then run on Waters Sep pak C18 (2 g) reversed-phase cartridge (70%: 30% H_2_0: MeOH) to further purify the active component. Finally, the active fraction (11 mg) was purified by C18 reversed-phase high-performance liquid chromatography (HPLC) using a CSC-Inertisil 150A/ODS2, 5μm 25 × 0.94 cm column with 1:3 MeCN/H_2_O as an eluting solvent with a flow rate of 2 mL min^−1^ to yield the major compound.

The melting point was measured on a Buchi melting point B-540 visual thermometer. The optical rotations were measured with a Bellingham-Stanley APD440 polarimeter. The UV spectra were recorded with Perkin Elmer UV−vis or Varian Cary 5000 UV−vis-NIR spectrophotometers. Infrared (IR) spectra were recorded on a PerkinElmer FTS FT-IR or Perkin Elmer Frontier Optica FT-IR spectrophotometers. ECD spectra were recorded on a JASCO J-815 spectrometer. The 1D and 2D NMR spectra were recorded on a 400 MHz Bruker FT-NMR Ultra Shield and 600 MHz Bruker AV-600 spectrometers in CDCl_3_ and/or acetone-d6 (CO) 206.2 and (CH_3_) 30.6, with TMS as the internal standard. Chemical shifts are reported in parts per million (δ), and coupling constants (J) are expressed in hertz. ESI-QIT-MS and HR-EI-MS spectra were measured on a Bruker-Hewlett- Packard 1100 Esquire-LC and a MAT 95 XL mass spectrometer, respectively. HPLC was performed on a Waters 515 HPLC pump system liquid chromatography using the following columns: RP C18 CSC-Inertsil 150A/ODS-2 column (25 × 0.94 cm) and a CHIRALCEL OD-H column (4.6 × 250 mm). Quick column chromatography (QCC) and column chromatography (CC) were carried out on silica gel 60 H (5 − 40 μm, SiliCycle Inc.) and silica gel 100 (63 − 200 μm, SiliCycle Inc.), respectively. Sephadex LH-20 was also used for CC in determining elutes of different size fractions. Precoated plates of silica gel 60 F254 were used for TLC analysis.

### Screening for anti-inflammatory activity

For cell-based THP-1 cytokine-release assay; the human monocytic cell line THP-1 (American Type Culture Collection, Manassas, Va.) was maintained in Roswell Park Memorial Institute, USA (RPMI) supplemented with 2 mM of l-glutamine, 100 U/mL penicillin, 100 mg/mL streptomycin, 25 µL of 12% (v/v%)4-(2-hydroxyethyl)-1-piperazineethanesulfonic acid, as the zwitterionic buffering agent (HEPES), and 10% (v/v%) foetal bovine serum (FBS). Induction of cell differentiation was done with 100 µL of 18% (v/v%) para-methoxyamphetamine (PMA) for 24 h. After incubation, non-adherent cells were removed by aspiration, and the adherent cells were washed with RPMI three times. For cell stimulation, the cells were further incubated both with and without lipopolysaccharide (LPS) for 24 h in fresh complete medium with 10% FBS. After cell plating, the test compounds dissolved in 0.5% DMSO were added to each well, and the plate was incubated for 30 min at 37.8°C. Finally, 20 mL (10mg/mL) of LPS per well was added, to obtain a final concentration of 1 mg/mL.

Blood was withdrawn from human volunteers and aliquoted to 96-well plate (50 µL each). Immunomodulator was then added (10 µL), incubated at 37°C, 5% O_2_ for 15 min before the addition of 10 µL *E. coli* (final concentration 10^5^ cells/mL). They were then incubated for 7 h, followed by the addition of 100 µL of PBS. After mixing, the plate was centrifuged at 1200 rpm for 5 min, and the supernatant was obtained and used for IL6 analysis. (Ukwatta et al. 2019)

### Detection of secreted cytokines – Α glucosidase activities

Human TNF-α and IL-6 were quantified by an enzyme-linked immunosorbent assay (ELISA) (Ukwatta et al., 2019). Briefly, 96-well ELISA plates (Maxisorp; Nunc, Naperville, Ill.) were coated with an antihuman TNF-α/IL-6 monoclonal antibody in a coating buffer (carbonate/bicarbonate buffer, pH 9.6), followed by overnight incubation at 48°C. The wells were blocked for 2 h, at room temperature with 10% FBS prepared in assay buffer. Biotinylated anti-human TNF-α/IL-6 polyclonal antibody was added, followed by avidin-horseradish peroxidase conjugate, which used tetramethylbenzidine as the substrate. The reaction was stopped by the addition of 2M H_2_SO_4_, and optical density was recorded in a Saphire microplate reader (Tecan) at 490 to 600 nm.

### Cytotoxic assay

HCT116 colon cancer cells (1 × 10^4^ cells/well) were cultured in a 96-well plate and allowed to adhere for 24 h at 37°C. The cells were treated with testing compounds (10 μM or μg/mL) in Dulbecco’s modified eagle’s medium (DMEM) for 24 h. The medium was removed and fresh DMEM containing 0.5 mg/mL of 3-(4,5-Dimethylthiazol-2-Yl)-2,5-Diphenyltetrazolium bromide (MTT) solution was added to each well for 2 h. After that, the medium was discarded by an aspirator. The violet formazan crystals in the viable cells were dissolved in 100 μL of DMSO. The absorbance of each well was read at a wavelength of 570 nm using a microplate reader. Doxorubicin was used as a positive control with an IC_50_ value of 9.74 μM. Percentage cell viability and percentage cytotoxicity were calculated by using the following equations.

$$\%cell\;viability=\frac{absorbance\;of\;treated\;well}{absorbance\;of\;control\;well}\times \;100$$

%cytotoxicity=100−% cell viability

### Screening for anti-filarial assay materials and mechanisms

Primary evaluation: *In vitro* assays: Based on viability of the parasites, *in vitro* motility and 3-(4, 5-dimethylthiazol-2-yl)-2,5,diphenyltetrazolium bromide (MTT) reduction assays were carried out using ursolic acid (UA), ivermectin (IVM) and diethylcarbamazine-citrate (DECC) as reference drugs. For incubation of microfilaria (mf) and adult worms cell culture plates (Nunc, Denmark) were used with Hank’s balanced salt solution (HBSS) of pH 7.2.

The UA and IVM were dissolved in DMSO, whereas 2 mg of DECC was dissolved in 10 mL of sterile triple-distilled water (STDW). The anti-filarial agents were used at twofold serial dilutions ranged from15.63–1000 mM (DECC), 1.56–100 mM (UA) and 0.31–20 mM (IVM). The final concentration of DMSO in the incubation medium was kept below 0.1%to ensure maintaining buffer capacity of test agent’s solution as control assay.

Motility assay: Efficacy of UA and reference drugs was assessed in vitro on microfilaria and adult worms of *B. malayi* (as target parasites) using motility (microfilaria and adult parasite) and MTT (adult parasite only) reduction assay. Duplicate wells containing 40–50 mf/100 ml/well (of 96-well plates) and 1 female worm/ml/well (of 48-well plate) were used. UA (100 mM) or reference drugs IVM (20 mM), or DECC (1000 mM) were added accordingly based on the mf motility indices to duplicate wells and incubated. The wells with a test compound and DEC were incubated for 24 h and those with IVM were incubated for 24 and 48 h as it has a slow action on the parasites. All incubations were at 37°C in 5% CO_2_ atmosphere. The effect on the motility of the parasite stages was examined under a microscope and scored. The experiment was repeated twice. In case of microfilaria, only motility assay was performed.

Motility assessment: Parasite motility was assessed under a microscope after 24/48 h exposure to test substance and scored as: 0 = dead; 1–4 = loss of motility (1 = 75%; 2 = 50%; 3 = 25% and 4 = no loss of motility). Loss of motility is defined as the inability of the worms to regain pretreatment level of motility even after incubating in fresh medium without the test agent at 37°C for 1 h, and was expressed as percentage (%) inhibition of control.

MTT (formazan colourimetric assay for the viability of worms): The same female worms used in motility were then gently blotted and transferred to 0.1 ml of 0.5% MTT in 0.01 M phosphate-buffered saline (pH 7.2) and incubated for 1 h at 37°C. The formazan formed was extracted in 1 mL of DMSO for 1 h at 37°C and its absorbance was measured at 510 nm. The mean absorbance value obtained for treated worms was compared with the controls. The viability of the treated worms was assessed by calculating the inhibition percentage of motility and MTT reduction over DMSO control worms.

Criteria for assessment of *in vitro* hits: 100% inhibition in the motility of female adults, mf and 50% inhibition in MTT reduction ability of female parasites were considered acceptable anti-filarial (microfilaricidal/adulticidal) activity and picked up shifts and subjected to further testing *in vivo*.

Secondary evaluation – Determination of IC_50_: (the concentration at which the parasite motility was inhibited by 50%). The parasites were incubated with twofold serial dilutions from 1.56 to 100 (UA), 0.31 to 40 (IVM) and 15.63 to 1000 mM (DECC) using triplicate wells of the cell culture plate. Experiments were run in duplicate and incubations were carried out in replicates for 24/48 h as above. After incubation, inhibition in motility (mf and female worm) and MTT reduction potential of the parasites were assessed as above. The experiment was repeated twice.

Determination of cytotoxic concentration 50 (CC_50_): The cytotoxicity assay of the test substances was carried out as published in literature with some modifications (Ukwatta et al. 2019). Briefly, VERO Cell line C1008 (African green monkey kidney cells) was plated in 96-well plates (Nunc, Denmark) at 0.16106 cells/mL (100 ml per well) in DMEM supplemented with10% heat-inactivated FBS. A three-fold serial dilution of the test substances (starting from .20 x LC100 conc. of the test agent) in buffer was added. The plates with a final volume of 100 mL/well were incubated in 5% CO_2_ atmosphere at 37°C. After 72 h of incubation 10 mL of 0.025%, Resazurin in phosphate-buffered saline (PBS; pH 7.2) was dispensed as indicator for viability followed by an additional incubation for 4 h and the plate was then read in a fluorescence reader (Synergy HT plate reader, Biotek, USA) at excitation wavelength of 530 nm and an emission wavelength of 590 nm. The assay was run in replicates in each of two independent experiments.

Data of IC_50_ and CC_50_ fed to statistical software (SPSS) and were analysed as described in the literature by linear interpolation between the two concentrations above and below 50% inhibition. (Paige et al. 2011)

Selectivity Index (SI) of the samples was computed by the formula as:

$$SI=\frac{CC_{50}}{IC_{50}}$$

## Results and discussion

### Isolation of endophytic fungi

A total of 29 morphologically distinct endophytic fungi, 18 from leaves, 04 from stems and 07 from bark were isolated from the collected plant specimens.

### Identification of the fungus

The endophytic fungus was identified as *Aspergillus terreus* based on the DNA sequence of the ITS ribosomal RNA gene. The closest accession number obtained when the sequences were deposited in the GenBank was KP131622.

### Microbial activities of crude endophytic fungal extracts

The mean diameter (± Standard error (SE)) of the inhibition zones of the active crude fungal extracts in the antibacterial and antifungal bioassays are shown in Tables 1 and 2 respectively. Four of the extracts were active against Gram-negative bacteria tested (KUBGACJ-07, KUBGACJ-11, KUBGACJ-14 and KUBGACJ-21) at 25 µg/disc, while 09 fungal extracts inhibited the growth of at least one Gram-positive bacterial strains at 10 μg/disc. As mentioned in the literature, (Hugo 1998) the lesser potential activity towards Gram-negative bacteria would be due to the rigid, stable and complex cell wall structures in them. Among them KUBGACJ-15, KUBGACJ-16 and KUBGACJ-27 were only active against *S. aureus* and other six extracts were active against *S. aureus*, MRSA and *B. subtilis*. KUBGACJ-14 showed antibacterial activity against Gram-positive and negative bacteria at 25μg/disc concentrations and also on 10μg/disc, respectively, further anti-fungal activity at 50μg/disc; thus, this fungus was selected for the isolation of bioactive metabolites after large scale culturing. Two crude extracts, KUBGACJ-02 and KUBGACJ-05, exhibited antifungal activity against *F. oxysporum* (UBC 12,145), *R. microporus* (UBC 6314) and *C. albicans* (ATCC 90,028) only but KUBGACJ 14 showed antifungal activity against all tested five fungal strains by showing activities against *C. gloeosporioides* (UBC 3110) and *A. niger* (UBC 9214) as well. It was selected for sequential isolation of the bioactive compound. The three extracts that showed antifungal activity also showed activity against Gram-positive bacteria. Although antifungal activities of plant endophytic fungi have been reported occasionally, the occurrence of antifungal activities of plant endophytic fungi is not as common as antibacterial activities.

**Table 1.** Antibacterial activities of the crude extracts of endophytic fungi of the plant *Bruguiera gymnorrhyza.*

	Antibacterial activity – Gram-negative (mean diameter of the inhibition zone in mm ± SE)				Antibacterial activity – Gram-positive		
	*E. coli*		*P. aeruginosa*		MRSA	*B. subtilis*	*S. aureus*
Fungal strain	100 µg/disc	25 µg/disc	100 µg/disc	25 µg/disc	10 µg/disc		
KUBGACJ-02	04.1 ± 0.1	-	05.1 ± 0.3	-	04.3 ± 0.2	02.1 ± 0.2	03.4 ± 0.1
KUBGACJ-05	05.2 ± 0.3	-	-	-	02.3 ± 0.3	07.3 ± 0.3	-
KUBGACJ-07	07.3 ± 0.4	05.3 ± 0.3	03.3 ± 0.2	02.3 ± 0.1	03.2 ± 0.1	04.3 ± 0.4	02.6 ± 0.1
KUBGACJ-11	07.9 ± 0.1	05.3 ± 0.4	04.3 ± 0.1	02.3 ± 0.2	05.3 ± 0.2	03.1 ± 0.2	02.1 ± 0.2
KUBGACJ-14	20.3 ± 0.2	16.3 ± 0.4	20.7 ± 0.1	18.4 ± 0.2	08.3 ± 0.4	06.3 ± 0.2	07.1 ± 0.4
KUBGACJ-15	09.3 ± 0.3	-	-	-	-	-	06.3 ± 0.3
KUBGACJ-16	05.4 ± 0.6	-	-	-	-	-	04.2 ± 0.1
KUBGACJ-21	16.3 ± 0.2	12.3 ± 0.4	14.1 ± 0.1	10.5 ± 0.2	04.2 ± 0.1	03.3 ± 0.4	03.4 ± 0.3
KUBGACJ-27	-	-	-	-	-	-	06.3 ± 0.4
+ Control (1 µg/disc)	23.3 ± 0.3	23.6 ± 0.3	21.2 ± 0.4	23.4 ± 0.3	20.6 ± 0.3	21.6 ± 0.3	22.3 ± 0.4
- Control	-	-	-	-	-	-	-

**Table 2.** Antifungal activities (Qualitative)^a ^of the crude extracts (50 µg/disc) of endophytic fungi of the plant *Bruguiera gymnorrhyza.*

Fungal strain	*C. albicans*	*F. oxyporum*	*R. microporus*	*C. gloeosporioides*	*A. niger*
KUBGACJ-02	**+**	**++**	-	-	**+**
KUBGACJ-05	**+**	**+**	**+**	-	-
KUBGACJ-07	-	-	-	-	-
KUBGACJ-11	-	-	-	-	-
KUBGACJ-14	++	+++	++	+++	+++
KUBGACJ-15	-	-	-	-	-
KUBGACJ-16	-	-	-	-	-
KUBGACJ-21	-	-	-	-	-
+ Control (1 µg/disc)	**++++**	**++++**	**++++**	**+++**	**+++**
- Control	-	-	-	-	-

aKey – + – inhibition zone <3 mm, ++ – inhibition zone <5 mm, +++ – inhibition zone <7 mm, ++++ – inhibition zone <9 mm, (-) – No inhibition

### Isolation and structure elucidation of the major compound (KUBGACJ-14)

**Figure 1.**
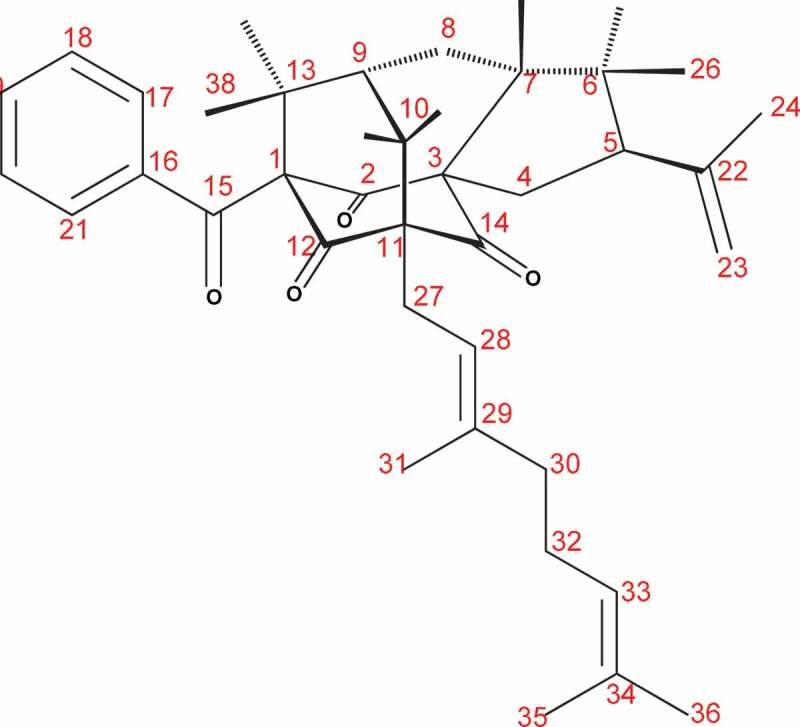
Structure of cowabenzophenone A

The bioassay-guided fractionation of the EtOAc extract led to the isolation of the active compound (4.3 mg) as a colourless crystalline solid eluting at a retention time of 37 min under the HPLC conditions (60%:40% H_2_0:MeOH) used. This compound showed a molecular ion peak at [M]^+^*m/z* 568.3547 in HR-EI-MS corresponding to the molecular formula of C_38_H_48_O_4_ (Figure 1). Analysis of ^1^H and ^13^C NMR data as well as 2D NMR (COSY, HSQC, HMBC, tROESY and HSQC ^15^N) spectral data in CDCl_3_ revealed that the structure of the active compound to be cowabenzophenone A matching with the data reported in the literature.^23^

The UV spectrum of the compound had maximum absorbance readings at 207, 242, 282 and 308 nm, while the IR spectrum had absorption bands corresponding to three carbonyl groups (1736, 1703 and 1686 cm^−1^). The basic skeletal structure determination was performed using the COSY, HMQC and HMBC spectra (Table 3). The sequence of C-7 to C-10 was confirmed in the COSY spectrum by the cross-peaks. Thus, the structure of Figure 1 was identified as cowabenzophenone A.

### Antimicrobial activity of cowabenzophenone a

Cowabenzophenone A showed strong selective antibacterial activities against Gram-positive *B. subtilits* (UBC 344), and *S. aureus* (ATCC 43,300) with MIC values of 1 and 2 μg/mL, respectively. It also showed promising activity against MRSA (ATCC 33,591), as 4 μg/mL. From the MIC assay it showed activity against Gram-negative *E. coli* (UBC 8161) and *P. aeruginosa* (ATCC 27,853), as 4 and 2 μg/mL, respectively (Table 4). Cowabenzophenone A also showed potential activities against the pathogenic fungi *C. albicans* (ATCC 90,028) *C. gloeosporioides* (UBC 3110) and *A. niger* (UBC 9214), 4, 2 and 2 μg/mL, respectively. Polymixin B, amphotericin and rifamycin were used as positive controls and their respective activities are shown in Table 4.

**Table 3.** ^1^H and ^13^C NMR data of cowabenzophenone A

	Cowabenzophenone A				
Position	δ_H_		δ_C_		HMBC (^1^H^13^C)
1	-		82.5	-	
2	-		203.8	-	
3	-		72.4	-	
4a	2.89 (t)		34.0	2, 3, 5, 14, 22	
4b	2.07 (m)			2, 3, 5, 14	
5	3.03 (dd)		56.0	6, 22, 23	
6	-		44.6	-	
7	2.20 (m)		55.0	2, 14	
8a	2.07 (m)		22.6	10, 13	
8b	1.76 (m)			3, 6, 7, 10, 13	
9	2.07 (m)		42.7	1, 7, 11	
10a	2.51 (dd)		35.1	8, 9, 11, 12, 27	
10b	2.20 (m)			11, 12	
11	-		67.9	-	
12	-		204.5	-	
13	-		47.5	-	
14	-		205.0	-	
15	-		193.2	-	
16	-		135.2	-	
17	7.07 (d)		128.6	15, 19, 20	
18	7.27 (t)		128.5	16, 17	
19	7.39 (t)		132.4	17, 18	
20	7.27 (t)		128.5	16, 17	
21	7.07 (d)		128.6	15, 19, 20	
22	-		144.5	-	
23a	4.90 (s)		113.2	5, 24	
23b	4.77 (s)				
24	1.77 (s)		23.7	22, 23	
25	0.87 (s)		26.7	5, 6, 7, 26	
26	1.01 (s)		26.7	5, 6, 7, 25	
27	2.62 (m)		29.2	11, 12, 28, 29	
28	5.29 (t)		119.1	27, 30, 31	
29	-		139.1	-	
30 31 32 33 34 35 36 37 38	2.07 (m) 1.66 (s) 2.07 (m) 5.06 (m)- 1.66 (s) 1.59 (s) 1.39 (s) 1.41 (s)		40.2 16.5 26.0 124.3 132.3 25.9 17.8 22.6 25.3	28,33 28, 29, 30 29, 30, 34 32, 36 – 33, 34 33, 34 1, 9, 13 1,9,13

**Table 4.** MIC values obtained for cowabenzophenone A and for positive controls

Compound name		Minimum inhibitory concentration values (MIC) – µg/mL					
	*B. subtilis*	*S. aureus*	*E. coli*	MRSA	*C. albicans*	*P. aeruginosa*	*C. gloeosporioides*
Cowabenzophenone A	**1**	**2**	**4**	**4**	**4**	**2**	**2**
Polymixin B	4	-	1	-	-	1	-
Amphotericin	-	-	-	-	1	-	1
Rifamycin	-	1	-	1	-	-	-

### Anti-inflammatory activities of cowabenzophenone a

This compound showed a value of 37% (results were expressed as percentages of IL6 relative to *E. coli*-stimulated blood, therefore, lower the number showed greater anti-inflammatory activity.) This compound showed an IC_50_ of 12.1 µg/mL.

### Alpha-glucosidase activities

This compound showed an α-glucosidase inhibitory activity exhibiting an IC_50_ value of 7.8 ± 0.5 μM.

### Cytotoxicity of cowabenzophenone a

This compound showed a cell viability value of 32%, resulting the cytotoxicity to be 68%. It showed cytotoxicity against HCT 116 colon cancer cell line with IC_50_ values of 10.1 μM.

### Anti-filarial activities of cowabenzophenone a

This compound showed considerably high potential anti-filarial activity due to its significantly low MIC, IC_50_ and LC_50_ values 0.358 ± 0.02 mg/mL, 0.708 ± 0.021 mg/mL and 3.89 ± 0.18 mg/mL, respectively, in comparison to the standard drug Ivermectin (IVM) (MIC, IC_50_ and LC_50_ values 3.12 ± 0.78 mg/mL, 6.25 ± 0.14 mg/mL and 16.57 ± 0.21 mg/mL, respectively) against microfilariae and adults. This compound showed a 50% inhibitive cytotoxic concentration value (CC_50_) of 0.521 mg/mL. Therefore, the calculated selectivity index (SI) of this compound was 69.

## Conclusions

Cowabenzophenone A, first described in December 2013, was isolated in a sequence of bioassay-guided fractionations, as a crimson crystalline solid. The respective structure elucidation was completed by spectroscopic techniques, which was shown to have a molecular formula of C_38_H_48_O_4_. This is the first report of extraction of cowabenzophenone A, from a mangrove endophytic fungus *Aspergillus terreus* isolated from a *Bruguiera gymnorrhyza* mangrove plant.

This is also the first publication of antibacterial, anti-fungal, anti-inflammatory, anti-filarial, alpha-glucosidase inhibitory and cytotoxic activities of cowabenzophenone A. Due to the promising biological activities exhibited of inflammation and alpha-glucosidase inhibitions, the compound can be further investigated to develop potential drug leads.
